# Stress-induced translocation of the endoplasmic reticulum chaperone GRP78/BiP and its impact on human disease and therapy

**DOI:** 10.1073/pnas.2412246122

**Published:** 2025-07-23

**Authors:** Amy S. Lee

**Affiliations:** ^a^Department of Biochemistry and Molecular Medicine, Keck School of Medicine, University of Southern California, Los Angeles, CA 90033; ^b^Norris Comprehensive Cancer Center, Keck School of Medicine, University of Southern California, Los Angeles, CA 90033

**Keywords:** GRP78/BiP, ER stress, cancer, chaperone, translocation

## Abstract

Since their discoveries in the 1960s as a family of proteins produced by cells in response to stress, molecular chaperones are increasingly recognized as major regulators of cellular homeostasis in health and disease. Among the heat shock protein 70 family, the 78-kDa glucose-regulated protein (GRP78), also referred to as BiP and encoded by the *HSPA5* gene, contains a signal peptide targeting it into the endoplasmic reticulum (ER). Through its interaction with the transmembrane ER stress sensors, GRP78 acts as a master regulator of the Unfolded Protein Response (UPR) which allows cells to adapt to stress observed in many human diseases. The discovery that ER stress not only upregulates GRP78 to cope with ER protein quality control but also actively promotes its relocation to other cellular compartments where they vastly expand its functional repertoire beyond the ER represents a paradigm shift. This Perspective describes the origin and linkage of GRP78 to the UPR and the mechanisms whereby ER stress actively promotes export of GRP78 from the ER, as exemplified by its translocation to the cell surface where it acts as a multifaceted receptor and a conduit for drug and viral entry, as well as its translocation into the nucleus, where it assumes the surprising role of a transcriptional regulator whereby reprogramming the cell’s transcriptome. Furthermore, this Perspective addresses how these and other atypical localizations of GRP78 impact human disease, with emphasis on cancer and COVID-19, and the exciting prospect that drugs targeting GRP78 could dually suppress tumorigenesis and viral infections.

## The Discovery of 78-kDa Glucose-Regulated Protein (GRP78) and Its Link to the Unfolded Protein Response (UPR)

Scientific pursuits can be unpredictable, but they are most thrilling when the unexpected turns into serendipitous breakthroughs opening new frontiers, changing paradigms with therapeutic potentials to benefit mankind. The story of the glucose regulated proteins (GRPs) began in the mid-1970s when researchers noticed that rat and chick embryo fibroblasts transformed by various strains of Rous Sarcoma viruses showed a significant increase in two membrane associated proteins around 75 and 90 kDa (1). The proteins were originally thought to be transformation-specific. However, in 1977, Ira Pastan of the National Cancer Institute made the pivotal determination that these were constitutively expressed cellular proteins and their induction was not the consequence of cell transformation, but rather a secondary effect of rapid depletion of glucose from the growth medium of transformed cells. Hence, they were named GRPs (2). The two most common GRPs in mammalian cells are GRP78 and GRP94 with molecular masses of 78 to 80 and 94 to 100 kDa, respectively, with GRP78 also commonly referred to as the binding immunoglobulin protein BiP (3). Further research revealed that these same proteins were also inducible by various agents or mutations that perturb cellular glycosylation, pH, oxidative state, and calcium homeostasis, suggesting that these proteins might not be simply mediating glucose transport or utilization (4–8).

So, how are GRP78 and GRP94 coordinately upregulated under diverse conditions and what are the functions of these proteins? Around the same time when the GRPs were first detected, the ability to clone individual genes from mammalian cells ushered in a new era of molecular biology. Despite advances in understanding how genes were regulated via operons in prokaryotes, how nonadjacent eukaryotic genes could be coordinately upregulated remained a nascent field. Amy Lee, then a graduate student at the California Institute of Technology, was fascinated by gene cloning technology and further inspired by the concept of gene regulatory networks pioneered by her postdoctoral mentor Eric Davidson at Caltech. Thus, in 1979 when offered a faculty position at the University of Southern California, she started to search for a model system in mammalian cells to study how genes that were not physically linked could be coordinately regulated. Her idealism inspired her to look for a novel system which could have the added advantage that there would be little competition. In her quest, she came across a letter to Nature in 1978 by Jose Melero and Alan Smith of the Imperial Cancer Research Fund, United Kingdom that described potential transcriptional control of three specific proteins of 94, 78, and 58 kDa of unknown identity at the nonpermissive temperature in a mutant Chinese hamster K12 cell line (9). Seizing upon this opportunity to uncover the mystery of the K12 system as a cornerstone of her new laboratory and her first NIH grant, she contacted Melero who generously supplied her with the K12 cell line to pursue the molecular angle. NIH also liked the novelty, so the stars were aligned for the start of her four-decade-long odyssey tackling the regulation and function of the GRPs.

In the era before genomics and PCR, one needed to obtain transcripts/complementary Deoxyribonucleic Acid (cDNA) to create probes to locate the gene to then find the regulatory elements. It turned out that K12 cells with the transcripts of the mystery proteins expressed in high abundance (about 1% of total cytoplasmic polyA^+^RNA) at 40.5°C compared to 35°C was an ideal system for the cloning of their genes. The first milestone was achieved when in 1981 Lee’s team succeeded in making a high-quality cDNA library of K12 cells at 40.5°C and from there identified the cDNA clones encoding for the 78- and 94-kDa proteins induced in the K12 cells (10). Following up on a tip from Melero that the molecular sizes of the K12 proteins inducible at 40.5°C were highly similar to the GRPs reported by Pastan, the Lee lab quickly determined that the K12 proteins were also inducible by glucose deprivation, hence, serendipitously cloned the first mammalian cDNA encoding for GRP78 and GRP94 (11–13). The K12 mutation was later determined to block the transfer of the oligosaccharide core from lipid carriers to nascent polypeptides in the endoplasmic reticulum (ER) (12, 14) and the 58-kDa protein inducible in the K12 mutant cell line is GRP58/ERp57, a member of the protein disulfide isomerase family (15). Utilizing the GRP cDNA and genomic clones containing the regulatory elements as probes, a model emerged that perturbations of glucose, calcium, and other stresses triggered elevated transcription of the *GRP* genes (8, 16), providing an early clue of the existence of a monitoring system that allows mammalian cells to sense such stresses and in response, initiate a transcriptional program in the nucleus as adaptive measures.

However, it remained unclear what the GRPs did and why they were induced by stress. In 1986, while searching for members of the heat shock 70-kDa (HSP70) protein family, Hugh Pelham of the Medical Research Council, United Kingdom, isolated an unusual cDNA clone p72 from normal rat liver which encoded a 72-kDa protein with a hydrophobic leader peptide and when expressed in COS cells was localized to the ER (17). Strikingly, the N terminus of mature GRP78 from the K12 cells determined through amino acid sequencing of the purified protein reported earlier by Lee (6) corresponded exactly to the predicted leader peptide cleavage site of p72, suggesting that p72 was rat GRP78. Through peptide mapping and immuno cross-reactivity, Pelham further proposed that GRP78/p72 was the same as the immunoglobulin heavy chain binding protein BiP abundant in antibody secreting cells facilitating in Ig assembly, and as a heat shock protein cognate facilitates the assembly of secreted and membrane bound proteins in general (3, 17, 18). In 1988, GRP78 and BiP were confirmed to be the same protein by direct amino-terminal sequence comparison and that posttranslational modifications of GRP78 were important in regulating its synthesis and binding to client proteins (19). In the same year, Mary Jane Gething and Joe Sambrook of the University of Texas Southwestern Medical Center demonstrated that the accumulation of misfolded proteins in the ER was the proximal signal that induced the synthesis of GRP78 and GRP94 (20). These successive discoveries linking stress response, chaperones, and ER homeostasis inspire a new research field on the UPR, which has a profound impact on our understanding of organellar stress signaling pathways, leading to an explosion of new research directions toward understanding the molecular mechanisms and therapeutic potential.

The UPR is an intracellular quality control system that is evolutionarily conserved in eukaryotic cells, including plants and activates intracellular signal transduction pathways integrated to adapt to demands imposed by ER stress (21, 22). The protective mechanisms of the UPR include increasing production of chaperone proteins such as the GRPs, transient arrest of translation, and degradation of misfolded secretory and membrane proteins. The UPR supports plant development and defense, protecting them from environmental stresses and pathogens, as well as responses to abiotic stress (22). In humans, the UPR is implicated in both health and disease, as it can protect normal organs against proteotoxic stress, but can also be leveraged by cancer cells, enabling aggressive growth or dormancy, as well as acquiring resistance to therapy (23–26). The remainder of this Perspective focuses on the stress induction of GRP78, a highly conserved protein across eukaryotes and a widely recognized benchmark of the UPR. GRP78 not only aids in correct folding of secretory proteins, regulates activation of the ER stress sensors, but also plays key roles in unanticipated pathways in and beyond the ER, which, in turn, exert wide influence in health and disease (15, 27–33).

## Intricate Mechanisms to Ensure Rapid and Strong Induction of GRP78 upon Stress

In 1988, with the isolation of the human gene encoding for GRP78 and the other GRPs in various species, the stage was set to decipher the genetic code for the coordinated induction of the *GRP* genes in mammalian cells (34). At the International Conference on the Dynamics and Regulation of the Stress Response held in Kyoto Japan in 1998, Amy Lee from the University of Southern California in the United States and Kazutoshi Mori from the HSP Research Institute, Japan, independently announced the discovery of the ER stress responsive element (ERSE). The ERSE sequence CCAAT(N_9_)CCACG, with N being a strikingly GC-rich region of nine base pairs, is both sufficient and necessary to confer ER stress induction of the mammalian GRPs. That was an exciting and gratifying moment considering that the two laboratories, working independently continents apart, reached the same conclusion simultaneously. The discovery of the ERSE made it possible to work backward and identify the molecular pathways whereby stress from the ER could be communicated to the nucleus, leading to the transcriptional activation of UPR targets and then, step by step, found the key players mediating the regulation (23, 35–37).

The induction of GRP78 is primarily regulated at the transcriptional level. Mammalian GRP78 is encoded by a single *HSPA5* gene, and its homologous deletion leads to embryonic lethality at day 3.5 (34, 38). Interestingly, plants often have multiple genes encoding for GRP78, with *Arabidopsis thaliana* containing three (39). This gene expansion may reflect functional diversification related to stress resilience in plants. During stress, to ensure prompt upregulation of GRP78, nature employs redundancy and backup support. Thus, the *GRP78* promoter contains multiple copies of the ERSE located upstream of the TATA element, with ERSE binding transcription factors including NF-Y, YY1, TFII-I, and Sp1, supplemented by an ERSE-independent pathway, mediated by activating transcription factor 4 whose translation is upregulated by the PERK arm of the UPR, in complex with CREB1 (40–42). A potent activator for *GRP78* induction is ATF6, a transmembrane ER sensor. Upon ER stress and release from luminal GRP78, ATF6 undergoes proteolytic cleavage to produce a nuclear form that binds the ERSE and activates transcription (43, 44). Thus, GRP78 is both a regulator and target of the UPR. Furthermore, single-molecule footprinting coupled with computational analysis have led to the discovery that the core *GRP78* promoter including its TATA box and transcription initiation site is constitutively depleted of nucleosomes, making it one of the first examples of a promoter of an active gene devoid of nucleosomes in mammals (45). This finding, coupled with the TATA box of the *GRP78* promoter being frequently occupied even in the noninduced state, guarantees a swift and robust upregulation of *GRP78* transcription upon ER stress, underscoring the importance of this gene in the ER stress response.

## Critical Roles of GRP78 in Cancer

Cancer cells are often subjected to ER stress triggered by both intrinsic and extrinsic factors such as altered cell metabolism, hypoglycemia, hypoxia, acidosis, viral infection, and genetic lesions, and GRP78 is commonly upregulated in a wide variety of cancers (30). The first clue that GRP78 induction is critical for tumorigenesis was obtained in a 1996 study showing that fibrosarcoma cells incapable of inducing GRP78 were impaired in tumor formation (46). Subsequently, through the creation of a floxed *Grp78* mouse where the *Grp78* allele could be partially, completely, or inducibly knocked out in specific tissues (38), it was revealed that *Grp78* haploinsufficiency was able to suppress Phosphoinositide 3-Kinase/Protein Kinase B (PI3K/AKT) activation mediated by the loss of the tumor suppressor gene *PTEN,* blocking prostate tumorigenesis and leukemogenesis yet had no effect on normal organs (47, 48). For pancreatic cancer, *Grp78* haploinsufficiency suppressed AKT, S6, extracellular signal-regulated kinase (ERK), signal transducer and activator of transcription 3 activation, and reduced epidermal growth factor receptor (EGFR) protein level critical for acinar-to-ductal metaplasia initiation, a key step in pancreatic tumorigenesis (49). Likewise, *Grp*78 haploinsufficiency suppressed mutant *Kras*-driven lung tumorigenesis at least in part through activation of UPR induced cell death (50), and targeting GRP78 suppressed KRAS protein expression but not SRC proto-oncogene, nonreceptor tyrosine-protein kinase (SRC), or p85 and reduced viability of cancer cells bearing various KRAS mutations in lung, colon, and pancreatic cancer (51). In addition, inhibition of GRP78 upregulated the translational inhibitor 4E-BP1, leading to reduced expression of MYC, the most deregulated oncoprotein in cancer, and loss of viability of oncogenic MYC tumors (52). Thus, through extensive cell-based, three-dimensional cultures and in vivo studies, GRP78 has emerged as a key promoter of cell proliferation, oncogenic signaling, angiogenesis, stemness, metastasis, and immune evasion and its induction in a wide range of cancer fuels tumor growth and spread, as well as therapeutic resistance (29, 30, 33, 53, 54). Nonetheless, this raises the intriguing question on how GRP78, an ER luminal protein, can affect such a wide array of pathways outside the ER to exert its influence on oncogenesis. As science is full of surprises, the answers may lie in the unexpected findings below, as summarized in *SI Appendix*, Fig. S1.

## Mechanisms for Active Translocation of GRP78 to the Cell Surface

Traditionally, GRP78 has long served as the very definition of an ER resident protein since the discovery by Pelham in 1986 through GRP78 that its Lys-Asp-Glu-Leu (KDEL) C-terminal peptide shared among other luminal ER proteins served as the ER retention signal (55). Thus, when GRP78 arrives at the Golgi, the KDEL receptor (KDELR) recognizes KDEL and packages it into coat protein complex I vesicles leading to Golgi-to-ER retrograde transport. But what if its ER localization is not absolute? An early hint that GRP78 could escape from the ER to the cell surface and regulate signaling came from a 2002 report by Salvatore Pizzo at Duke University. While purifying α_2_-Macroglobulin (α_2_-M) cell surface membrane binding proteins in human prostate cancer cells, GRP78 was identified as the predominant binding protein and was essential for α_2_-M signal transduction (56). Remarkably, in 2003, global profiling of cell surface proteome of a variety of cancer cells identified chaperone proteins including GRP78, other GRPs, and HSPs to be highly abundant on the cell surface (57). Around the same time, Renata Pasqualini and Wadih Arap at the University of Texas MD Anderson Cancer Center, through fingerprinting the circulating repertoire of antibodies from cancer patients, identified the cell surface form of GRP78 (cs78) as a molecular target expressed in metastatic tumors with potential as a conduit for cancer specific delivery of cytotoxic agents while sparing normal tissues (58, 59). Furthermore, cs78 formed complex with the human plasminogen Kringle 5 on the surface of proliferating endothelial cells and stressed tumor cells and mediated its antiangiogenic and antitumor activity (60). GRP78 also formed complex with Cripto, a GPI-anchored protein, at the cell surface of multiple cell lines to inhibit transforming growth factor beta (TGF-β) signaling and enhance cell growth (61). Surprisingly, PAR-4, normally a cytosolic and nuclear protein, was found to spontaneously secrete, and cs78 was identified as receptor for extracellular PAR-4 to activate an extrinsic pathway for apoptosis in cancer cells (62).

Despite these important findings, it has been intriguing to many in the field as to why an ER resident luminal protein containing the KDEL retrieval motif can escape the ER and why is it found on the cell surface. A simple guess is that the overload of GRP78 in stressed cells overwhelms the KDEL retrieval mechanism, thereby allowing its leakage to the cell surface via the ER-Golgi secretory pathway. On the other hand, ER stress could activate specific mechanisms to promote relocalization of GRP78 and other ER chaperones to the cell surface to allow the cells to expand their functions beyond the ER. If true, this would be paradigm shifting in our understanding of the role of chaperones in health and disease. In support of active transport to the cell surface rather than passive leakage, a 2010 study revealed that in human HEK293T cells while the total GRP78 level increased by about threefold following ER stress, there was a 12-fold increase in cell surface GRP78 compared to nonstressed cells (63). Importantly, additional kinetics studies showed that ER stress rapidly induced cell surface localization of ER chaperones before an increase in their intracellular levels (64). So, how is that achieved?

The proto-oncogene SRC, which is overexpressed and commonly activated in human malignancies, is known to regulate proliferation, invasion, adhesion, and survival. Interestingly, active SRC has been reported to localize to the ER and Golgi complex and inhibit retrograde transport of resident ER proteins containing a COOH-terminal KDEL sequence by dispersing the KDELR from the Golgi complex to the ER with no effect on anterograde transport (65). In 2018, Lee and her team discovered that in addition to being activated by ER stress, SRC actively promoted cell-surface relocalization of ER chaperones in a variety of solid and blood cancer cell lines, and importantly, SRC was both necessary and sufficient for this process (64). Upon ER stress, SRC formed a complex with the ER stress transmembrane sensor inositol-requiring enzyme 1 alpha and was activated through Y419 phosphorylation. This led to ASAP1 phosphorylation and Golgi accumulation of ASAP1 and Arf1-GTP, resulting in dispersion of the KDELR from the Golgi and suppression of Golgi-ER retrograde trafficking, thereby allowing a small subfraction of GRP78 (about 4%) and other luminal ER chaperones to relocalize to the cell surface (*SI Appendix*, Fig. S1*A*) (63, 64). Given the high abundance of GRP78 in the ER of stressed cells, the small amount of cs78 could easily elude detection and was missed for nearly two decades. Nonetheless, the ER to Golgi complex escape path is not obligatory for all cancer cells. For example, in colon and lung cancer cells, upon ER stress, ER luminal chaperones could bypass the Golgi and unconventionally traffic to the cell surface via endosomal transport by membrane fusion between ER-derived vesicles, and this process was mediated by Rab GTPases, the cochaperone DNAJC3 and regulated by ER-stress induced PERK-AKT-mTOR signaling (*SI Appendix*, Fig. S1*A*) (66). Additionally, other UPR sensors could also facilitate the ER escape mechanism, as the exposure of calreticulin on the cell surface has been reported to depend on PERK in immune cells (67). Interestingly, cell surface GRP78 localization required its substrate binding domain (68). Thus, GRP78, through its interaction with client proteins in the ER, could cotraffic with them to the cell surface, as exemplified by GRP78 interacting with the transmembrane protein CD44 in the ER as well as on the cell surface of breast cancer cells (69).

Once GRP78 reaches the cell surface, how does it anchor there? While it was thought that GRP78 contained three hydrophobic regions that allowed it to embed into membranes, analysis of the primary GRP78 amino acid sequence by the TMpred program identified only the ER signal sequence with an unsurprisingly significant score. In contrast, the scores of the three hydrophobic regions within the mature GRP78 protein were below the threshold of significance, suggesting that it is not a traditional transmembrane protein. In 2015, a comprehensive analysis of the soluble and membrane embedded forms of intracellular and cell surface GRP78 revealed that GRP78 primarily exists as a peripheral protein on the plasma membrane via interaction with other cell surface and GPI-anchored proteins (*SI Appendix*, Fig. S1*A*), with endogenous GRP78 readily detected at the plasma membrane of stressed human colon cancer cells, as imaged by single molecule, superresolution total internal reflection fluorescence microscopy (68). GRP78 also colocalized with CD44 on the surface of breast cancer patient-derived circulating tumor cells, and superresolution dual-color single particle tracking further revealed dynamic interaction and coconfinement of GRP78 and CD44 in the plasma membrane nanodomains of breast cancer cells (69). Collectively, these studies firmly established the existence of GRP78 on the surface of live cells.

## Functional Significance of Cell Surface GRP78 in Cancer

The discovery that upon stress GRP78 translocates to the cell surface and is anchored there via interaction with cell surface proteins opens a new research frontier in deciphering the various binding partners of GRP78 on the cell surface in different cell types regulating a wide variety of signaling pathways. As this important topic has been extensively reviewed (27, 33, 70–72), the section below highlights some mechanisms whereby cs78 promotes cancer proliferation, survival, and metastasis. The PI3K/AKT pathway is activated in a wide array of cancers, leading to proliferation and therapeutic resistance. Cs78 forms complex with both the regulatory (p85) and catalytic (p110α) subunits of PI3K on the surface of cancer cells, resulting in stimulation of phosphatidylinositol (3,4,5)-trisphosphate production and elevated PI3K activity (73). On the other hand, TGF-β signaling is a potent inducer of growth arrest in cancer cells, and the GPI-anchored cell surface protein CD109 is a known negative regulator of TGF-β signaling by suppressing Smad2/3 phosphorylation and promoting degradation of the TGF-β receptor. On the cell surface, GRP78 binds to and acts in concert with CD109 to promote routing of the TGF-β receptor to the caveolae for degradation, thereby disrupting activation of Smad2 and TGF-β mediated growth arrest (64). For cancer invasion, CD44 is a type I transmembrane glycoprotein known to facilitate cell adhesion and migration in a variety of cancers. In tamoxifen-resistant breast cancer, cs78 binds to a variant form of CD44 on the cell surface and stabilizes it, as treatment with antibodies against GRP78 reduces cell surface CD44 protein levels, leading to F-actin disorganization and loss of cell attachment and spreading (69). Moreover, while tumors express many antigens, cs78 can serve as an autoantigen that triggers the production of anti-GRP78 autoantibodies, which upon binding to cs78 induces inositol triphosphate-mediated ER Ca^2+^ release, leading to promotion of tissue factor (TF) procoagulant activity, which in turn promotes tumor growth and metastasis (74).

## Translocation of GRP78 to the Nucleus and Its Nuclear Function

In early 2000s, GRP78 was detected in yet another unexpected compartment, the nucleus, when overexpressed or induced by ER stress and cross-linked to DNA in irradiated cells (75, 76). The mechanism and the role of nuclear GRP78 remained largely a mystery until 2023, while investigating how GRP78 knockdown suppresses EGFR expression in lung cancer, Lee and her team discovered that GRP78 regulated *EGFR* transcription and that it contained a nuclear localization signal critical for its entry into the nucleus (77). Queries of the human reference protein interactome mapping database identified a nuclear transcriptional repressor protein ID2 as a top binding partner of nuclear GRP78, which was confirmed via biochemical and imaging assays in cells. In nonstressed cells, ID2 is known to bind to E-protein and prevents its interaction with basic helix–loop–helix transcription factor. The same study revealed that under stress, GRP78 was upregulated and a small subfraction (about 2 to 5%) translocated to the nucleus where it bound and sequestered ID2, relieving its inhibitory effects on transcription leading to activation of genes and pathways, notably those important for migration and invasion (*SI Appendix*, Fig. S1*A*) (77). Subsequent studies determined that the vast majority of nuclear GRP78 was the mature form devoid of its ER signal peptide, and both its ATPase and substrate binding activities were required for its transcriptional regulatory activity (78). Interestingly, GRP78 and GRP94 are known components of chaperone complex in the ER (79). It turned out GRP94 also contains a nuclear localization signal and colocalizes with GRP78 in the nucleus (78). The same study also discovered defects in ER-associated protein degradation (ERAD) implicated in neurodevelopmental disorder, adaptive immunity and tumor resistance, not only did not impede, but rather enhanced GRP78 translocation into the nucleus. Furthermore, it was recently reported that GRP78 formed complex with the transcription factor HIF-1α in glucose-deprived pancreatic cancer cells, and through binding to the promoters of the target genes activated transcription and promoted metabolic reprogramming (80). Collectively, these findings carry the important implication that nuclear GRP78, through interaction with factors regulating the transcriptional machinery, or other mechanisms, can potentially impact many cellular functions in health and disease. These discoveries usher in a new frontier for nuclear GRP78 research yet to be explored.

## Other ER-Stress Induced Relocalization of GRP78 and Its Function

ER-stress can also relocalize GRP78 to the mitochondria, cytosol and even be secreted in specific cell types to assume diverse new functions (*SI Appendix*, Fig. S1*A*) (27). For example, GRP78, by modulating ER-mitochondrial Ca^2+^ crosstalk and preserving mitochondrial membrane potential after stress, protects astrocytes against ischemic injury (81). At the mitochondria-associated ER membrane (MAM), GRP78 regulates steroidogenesis through regulating the intermediate folding of a key steroidogenic protein (82). In lung cancer, GRP78 in complex with mitochondrial proteins LETM1 and GRP75 is postulated to be important in MAM formation and mitophagy, thereby exerting mitochondrial quality control (83).

Fortuitously, in 2009, Lee and her team discovered that in leukemia cells ER stress induces alternative splicing (retention of intron 1) and alternative translation initiation of GRP78, generating a novel isoform GRP78va, which is devoid of the ER signaling peptide and is cytosolic, where it activates PERK signaling and enhanced leukemic cell survival (*SI Appendix*, Fig. S1*A*) (84). Interestingly, calreticulin, a luminal ER chaperone, has a cytoplasmic form arose from aborted translocation that regulated nuclear transcription (85). Furthermore, ER stress actively promotes a “protein reflux” system to deliver intact, folded ER proteins into cytosol without degrading them, and this system is enhanced when ERAD factors are crippled (86). The refluxed proteins could gain new function in the cytosol as exemplified by the refluxed cytosolic AGR2 protein binding and inactivating the tumor suppressor p53 (87). Some tumors secrete high levels of GRP78, which promotes tumor cell proliferation and blocks the antiangiogenic activity of bortezomib by binding to the cell surface receptor of endothelial cells and activates the ERK and AKT pathways (88). GRP78 was reported to be secreted via exosomes in colon cancer cells, and that increased GRP78 acetylation prevented its sorting into multivesicular bodies, decreasing its secretion and impairing tumor growth (89). Thus, stress-induced GRP78 translocation and secretion can vastly expand its functional repertoire and warrants vigorous investigation.

## Therapeutic Implications

GRP78 is commonly overexpressed in cancer and confers therapeutic resistance with its protective effect not only observed in proliferating cancer cells but also in dormant cancer cells, tumor-initiating cells as well as tumor-supporting stroma cells (29–31). Thus, GRP78 has emerged as an exciting target to combat cancer. Nonetheless, as GRP78 is an essential protein for cellular homeostasis, the key issue is whether a therapeutic window can be established allowing anti-cancer efficacy while minimizing toxicity. In support, both male and female *Grp78* heterozygous mice up to 2 y of age in different genetic backgrounds showed no impairment with respect to body weight, organ integrity, behavioral and memory performance, inflammation, and chemotoxic response (90). Furthermore, *Grp78* heterozygosity promoted adaptive UPR and attenuated diet-induced obesity and insulin resistance (91). In contrast, multiple studies showed that reduction of GRP78 by 50% in the whole body or specific tissues potently suppressed tumorigenesis while sparing normal organs (30). Hence, while most normal tissues can function with low basal level of GRP78, cancer progression requires high GRP78 levels. Since the topic of anti-GRP78 agents including both natural and synthetic agents with anticancer activities both in vitro and in vivo has been extensively reviewed recently (30, 33, 92, 93), the discussion below focuses on some exciting new developments, their promises, and challenges.

## Anticancer Small Molecule Inhibitors Targeting GRP78

Small-molecule agents that interfere with the synthesis, stability, or activity of GRP78 have the advantage that they can simultaneously shut down multiple progrowth and prosurvival pathways mediated by GRP78 in all cellular compartments, which will be difficult for cancer cells to overcome during the treatment period. Blocking the acute stress induction of GRP78 is attractive since higher level of GRP78 is required to fuel cancer growth and resistance. One such agent is a first-in-class ruthenium-complex BOLD-100 currently undergoing clinical Phase 2 evaluation for treatment of advanced gastrointestinal cancer in combination with chemotherapy (94). This agent, previously referred to as IT139/NKP-1339, was identified in 1989 in an anticancer drug screen and subsequently determined that GRP78 is a major target of its apoptotic activity (95). BOLD-100 triggered ER stress in a variety of cancer and resistant cell lines but not normal cells, and in xenograft studies suppressed GRP78 upregulation by BRAF inhibitor in the tumor with no effect on GRP78 expression in adjacent normal cells (95). How might BOLD-100 preferably affect GRP78 induction in cancer cells? One possibility is that BOLD-100 is primarily bound to albumin, which is highly abundant in serum and more highly taken up and metabolized by rapidly growing, nutrient-starved cancer cells.

Interestingly, a high-throughput screen of clinically relevant compound libraries yielded a surprising result that many of the top hits that block stress induction of GRP78 in cancer were cardiac glycosides, and among them, oleandrin (OLN) was the most potent, resulting in decreased expression of the ER, cell surface, and nuclear forms of GRP78 (96, 97). OLN at nanomolar concentrations blocked stress-induced loading of ribosomes onto *GRP78* mRNAs, enhanced apoptosis, sensitized colorectal cancer cells to chemotherapy, decreased viability of patient-derived colon cancer organoids, and impeded growth of aggressive breast cancer. Mechanistically, the OLN suppressive activity on GRP78 induction strictly depends on the integrity of the Na^+^/K^+^ ATPase α3 isoform, which has protein synthesis regulatory activity and since this isoform is preferably expressed in human cancer cells, this could partly explain the enhanced cytotoxicity of OLN in malignant cells compared to normal cells (97). Furthermore, PBI-05204 which contains OLN as its active ingredient and exhibited a safe profile in Phase 1/2 clinical trials in cancer patients, suppressed the growth of patient-derived glioblastoma stem cell spheroids in part through decreased GRP78 expression (98, 99). Thus, although agents such as BOLD-100 and OLN do not directly bind GRP78 and are not specific GRP78 inhibitors, their ability to block acute stress induction of GRP78 deprives cancer cells of the most potent prosurvival component of the UPR while exacerbating ER stress, a double disaster for the cancer cells.

HA15, identified in 2016 as a lead thiazole benzenesulfonamide compound in a screen for antimelanoma activities, selectively binds GRP78 and elicits ER-stress-induced cancer death through activating apoptosis and autophagy mechanisms (100). In addition to overcoming resistance to BRAF inhibitors in melanoma cells and EGFR-tyrosine kinase inhibitor resistance in non–small lung cancer cells, HA15 lowered viability of a wide range of cancer cells, including those bearing various *KRAS* mutations (51, 100, 101). Treatment of mice with HA15 showed no change in their behavior, body mass, or liver mass, suggesting an absence of hepatomegaly (100). BPR001-615, an optimized analog of HA15, is being developed as a first-in-class clinical candidate about to go into clinical trials for oral use to treat gastrointestinal cancers. YUM70, a derivative of 8-hydroxyquinone identified in 2021 in a phenotypic screen for cytotoxicity from 40,000 drug-like compounds, inhibited pancreatic cancer growth with no apparent toxicity to normal tissues, and subsequently determined that it directly binds GRP78, inactivating its function and triggering ER-stress mediated apoptosis (102). YUM70 synergized cytotoxicity with topoisomerase and histone deacetylase inhibitors in pancreatic cancer, reduced viability of head and neck cancer cells, and resensitized cisplatin-resistant cells (102, 103). Additionally, DX2-145, a GRP78 Proteolysis Targeting Chimera developed by incorporating YUM70, a linker and an E3-recruiting ligand, elicited partial degradation of GRP78 in cancer cells, suggesting the feasibility of this exciting new approach to eliminate GRP78 in the tumor (102). Collectively, the fact that independent, unbiased, large scale anticancer drug screenings led to the discoveries of GRP78 as their direct target validates GRP78 as critical for cancer cell viability. A comparison of the properties and strengths of the above agents is summarized in *SI Appendix*, Table S1.

Furthermore, regarding precision medicine, GRP78 as an abundant ER protein is easily detectable by immunohistochemical staining in patient tumor biopsies, and since GRP78 is detected in patient plasma, a noninvasive enzyme-linked immunosorbent assay test can also be used to screen for GRP78 levels and select patients most likely to respond to the drug. While the lack of standard high-throughput screening methods for specific GRP78 inhibitors could have hampered past progress for their identification, the emergence of AI/machine learning in the structural characterization of therapeutic targets in cancer (104) could vastly accelerate the discovery, evaluation, and optimization of small-molecule clinical candidates targeting GRP78 itself or its interaction with specific binding partners. This, coupled with recent advances in targeted drug delivery to cancer cells and their microenvironment, holds great promise for expanding the repertoire of anti-GRP78 therapy and limiting off-target effects.

## Anticancer Agents Targeting Cell Surface GRP78

The discovery that cs78 is preferably expressed in malignant cells and further increased in metastatic and drug-resistant tumors (73) offers the opportunity for cancer-specific therapy and drug delivery while sparing normal organs. As cs78 is detected in tumor-initiating cells, as well as in hypoxic endothelial cells that support tumor growth, cytotoxic agents against cs78 can dually target the tumor and its microenvironment (30). A murine anti-GRP78 monoclonal IgG antibody C107 directed against the C-terminal region of GRP78 induced apoptosis in melanoma cells and slowed their growth in mice (105). Notably, the C107 antibody exhibited higher reactivity to cs78 in ex vivo samples as compared to cells in vitro, suggesting that in vitro assays of cs78, commonly used in drug screens, may underestimate the in vivo cs78 levels and drug efficacy in vivo. MAb159, a high-affinity GRP78-specific mouse monoclonal IgG antibody potently suppressed PI3K/AKT signaling, tumor growth, metastasis, and synergized with topoisomerase inhibitor (106). A humanized MAb159 retained antitumor activity with favorable pharmacokinetics with no toxicity in mice and showed potential as an in vivo imaging agent for selection of patients expressing cs78 (106, 107). Despite these promising results, thus far, only a human monoclonal IgM antibody (PAT-SAM6), isolated from a gastric cancer patient and targeting a modified cs78, has been tested in Phase 1/2 clinical trial (108). While this antibody showed a safe profile and partial remission in a multi-drug-resistant multiple myeloma patient in combination therapy, IgM production is technically challenging. Thus, identification of its cs78 binding domain and its targeting via IgG or small molecules may offer a new approach toward its clinical application.

In contrast to the anticancer activities of GRP78 C-terminal antibodies, Richard Austin at McMaster University, Canada, reported in 2017 that binding of anti-GRP78 autoantibodies to the N-terminal region of cs78 promoted tumor growth and metastasis via activation of TF and that disruption of the cs78/anti-GRP78 autoantibody complex by heparin suppressed prostate cancer xenograft growth (74). Recently, Austin and his team used the AtomNet platform (Atomwise), an AI-enabled drug discovery engine, to perform a virtual high-throughput screen with a commercial library of over seven million small molecules and identified several “GRP78 binders” with the ability to mitigate anti-GRP78 autoantibody-mediated TF procoagulant activity in cultured endothelial cells (109). Future experiments will determine its applicability as novel therapies for cancer and other human diseases.

Leveraging their seminal discovery of cs78 as a tumor antigen in cancer patients in 2003, Renata Pasqualini and her team at MD Anderson Cancer Center devised therapeutic strategies including cs78 targeting peptides conjugated to proapoptotic peptide, and peptides on phage-based gene delivery vector capsid, enabling diagnostic imaging by positron emission tomography/computed tomography and drug-inducible cytotoxicity (58, 59, 110–112). These studies yielded promising antitumor effects in multiple cancer types and metastasis, suggesting broad translational efficacy. While an earlier cs78 targeting peptide exhibited toxicity observed only in nonhuman primates, it is associated with the peptide sequence, not GRP78 (113), and new peptide ligand has been identified that binds GRP78 in a screening using a PDX model of prostate cancer (112). Through the development of the Selection of Phage-displayed Accessible Recombinant Targeted Antibodies technology, novel recombinant antibodies against GRP78 have been identified, showing robust tumor localization, with demonstrated potential clinical utility for antibody-drug conjugates and gene therapy (114). In addition, the anti-GRP78 antibody can be engineered to be dual specific or pH-sensitive to further enhance tumor and tumor microenvironment specificity.

In 2006, Kim Janda at the Scripps Research Institute, CA, identified the cyclic 13-mer Pep42, CTVALPGGYVRVC as a powerful tool in the construction of drug conjugates to selectively kill malignant cancer cells (115). Cs78 is the cellular receptor for Pep42, which specifically internalizes into and kills cs78-expressing cancer cells. In 2022, while developing Chimeric Antigen Receptor T-Cell (CAR-T) cells for acute myeloid leukemia (AML), Paulina Velasquez and her team at St. Jude Children’s Research Hospital, TN generated for the first time a peptide-based GRP78 CAR, showing that GRP78 was expressed on the cell surface of primary AML blasts but not hematopoietic progenitor cells (HPCs), and CAR-T cells directed to cs78 via Pep42 exhibited robust anti-AML activity without HPC toxicity (116). Recent developments on a bispecific approach using a peptide-scFv antigen recognition domain for the CARs targeting cs78 expressing AML cells further showed efficacy in vitro and in vivo, with prolonged survival (117, 118). As cs78 is also highly expressed in multiple solid tumors including brain and pancreatic cancers, CAR-T cells directed to cs78 via Pep42, alone or in combination therapy, have been shown to selectively and efficiently curb tumor growth (119, 120). Collectively, these exciting new studies highlight the appeal of cs78 as GRP78-specific CAR therapy across cancers. Nonetheless, since tumor responses can be influenced by the biology of the specific disease targeted and normal tissues may transiently express a low level of cs78 following radiation or chemotherapy treatment, these and other issues warrant vigorous investigation using immunocompetent animal models to clear the hurdles to clinical trials.

## Critical Role of GRP78 in Viral Infection

It is well documented that viruses spanning many different virus families, upon infection of the host cells, trigger ER stress leading to the onset of the UPR and upregulation of GRP78 (121, 122) and ER chaperones play important roles in viral infections (123, 124). The discussion below focuses on two recent developments: 1) the relationship between GRP78 and severe acute respiratory syndrome coronavirus 2 (SARS-CoV-2), the causative agent of the COVID-19 global pandemic and 2) the exciting prospect of dually suppressing cancer and viral infections by targeting GRP78.

SARS-CoV-2 is enveloped by a lipid bilayer containing viral glycoproteins on its surface to bind to host receptors to facilitate entry. Since these membrane-embedded viral envelope proteins are synthesized in massive amounts in the ER, unsurprisingly, GRP78 is upregulated in SARS-CoV-2-infected human lung epithelial cells (*SI Appendix*, Fig. S1*B*) (125). Robust in situ GRP78 immunostaining was observed in autopsy analysis of lungs from COVID-19 patients, and higher serum GRP78 levels were detected in COVID-19 patients (126, 127). Likewise, single-cell profiling of Ebola Virus disease in vivo revealed that GRP78 is among the prominent prosurvival genes being upregulated in the infected cells (128). The discovery that viral infection induces GRP78 translocation to the cell surface to promote viral entry, as well as its involvement in subsequent viral assembly and egress advances GRP78 research in viral diseases with therapeutic implications.

## Cell Surface GRP78 Is a Critical Host Auxiliary Factor for SARS-CoV-2 Entry and Infection

An important early observation revealed that GRP78 was upregulated on the surface of the Middle East Respiratory Syndrome Coronavirus (MERS-CoV) infected cells and both MERS-CoV and bat coronavirus HKU9 utilized GRP78 as a coreceptor for attachment onto host cells and viral entry (129). In 2020, Abdo Elfiky and his team at Cairo University, Egypt performed a molecular docking analysis and identified a putative site of interaction between GRP78 and the receptor binding domain of the SARS-CoV-2 Spike protein (SARS-2-S) (130). Lee and her team at the University of Southern California utilized biochemical and imaging approaches to establish that GRP78, in fact, could form a complex with SARS-2-S, as well as with angiotensin-converting enzyme 2 (ACE2), the primary cellular receptor for viral entry, both on the cell surface and at the ER (*SI Appendix*, Fig. S1*B*) (131). Furthermore, knockdown of GRP78 or treatment of lung epithelial cells with the anti-GRP78 antibody MAb159 not only depleted cs78 but also reduced the cell surface level of ACE2, SARS-CoV-2 entry, and infection (131). These proof-of-principle studies provide direct evidence that cs78 is critical for SARS-CoV-2 infectivity and represents a potential target to combat this and a wide range of viral pathogens that utilize GRP78 for entry and production (123, 124, 131, 132).

Unexpectedly, while studying how β-coronaviruses including SARS-CoV2 exited the host cell, it was discovered that GRP78 and calreticulin, both ER luminal chaperones, were selectively cotrafficked with coronaviruses to lysosomes and coreleased with them outside the cell (133). Since GRP78 facilitates viral entry, maintaining GRP78 on the virion may help prime the virion to infect another cell. Likewise, GRP78 has been reported to comigrate and enhance infectivity of the Japanese encephalitis virus, a mosquito-borne flavivirus and a leading cause of deadly viral encephalitis in Southeast Asia (134) as well as promoting viral binding and internalization of other flaviviruses Dengue and Zika (132).

## GRP78 as Host Target for Dual Suppression of Viral Infection and Cancer

Considering that the putative Spike protein recognition site for GRP78 exhibits remarkable conservation among different SARS-CoV-2 strains, including the recent variants (97), agents targeting GRP78 can deprive the virus of an essential chaperone for viral entry and production, with the added advantage that GRP78 is a stable host protein in contrast to the rapidly evolving virus. Interestingly, the anti-GRP78 agents discussed in the cancer section above also exhibit broad antiviral activity. For example, BOLD-100, a small molecule suppressor of GRP78 stress induction, potently inhibits replication of SARS-CoV-2 and other evolutionarily divergent viruses HIV and human adenovirus type 5 (135). OLN, a cardiac glycoside that inhibits acute stress induction of GRP78, at nanomolar concentrations, suppressed GRP78, SARS-CoV-S, and N protein levels in the infected cells without compromising cell viability (97). OLN is effective against SARS-CoV-2 variants and enhances the efficacy of FDA-approved anti-COVID therapies Remdesivir and Nirmatrelvir. OLN also exhibits antiviral activity against a wide range of viruses, including the Ebola virus where its re-emergence could pose serious public health concerns (136).

HA15, an anticancer agent that specifically binds and inhibits GRP78 activity, blocks SARS-CoV-2 infection without affecting cell viability and substantially reduces the viral RNA load in the lungs of H18-hACE2 transgenic mice infected with SARS-CoV-2 (125). Furthermore, a recent study revealed that GRP78 is a proviral factor for diverse double-stranded DNA viruses and that HA15 exhibits a broad-spectrum antiviral activity with minimal toxicity for normal cells at concentrations higher than those used to block viral replication (137). Another anticancer GRP78 inhibitor YUM70 blocks viral entry mediated by the original and variant spike proteins, reduces SARS-CoV-2 infection without impairing cell viability, and ameliorates lung damage in mice infected with the virus, prolonging survival (138). Thus, while a primary concern when targeting host factors such as GRP78 for therapeutic antiviral intervention is the potential for cytotoxicity, collectively these studies, in combination with previous in vivo studies showing GRP78 haploinsufficiency in aged mice has no adverse effects on organ homeostasis in general (90), support the notion that GRP78 inhibition can be tolerable and manageable. Future drug screens to identify binders to GRP78 disrupting specific GRP78 interactions could further limit undesirable off-target effects.

## Conclusion

A wide range of cancer and viral infections upregulate GRP78 and hijack its functions at multiple locations, thus anti-GRP78 agents can be efficacious against cancer, COVID-19, and other human diseases that depend on stress induction of GRP78 for their pathological progression. Despite these advances, there remain major mysteries and challenges. Regarding mechanisms, most GRP78 expression studies were performed in cell cultures relying on nonphysiological stress inducers; thus, there is a pressing need to advance the field under physiological conditions in vivo. Regarding function, after nearly five decades since its discovery, GRP78 continues to surprise us, as exemplified by the roles of nuclear GRP78 which have yet to be fully explored. The involvement of GRP78 in other pathologies such as neurodegenerative and metabolic diseases not covered in this Perspective is emerging rapidly and deserves vigorous investigations. Finally, drug development on GRP78 is still in its early stages, so the most exciting phase of translation to the clinic has yet to be fulfilled.

## Supplementary Material

Appendix 01 (PDF)

## Data Availability

There are no data underlying this work.
