# Supplementary material for: Stress-induced translocation of the endoplasmic reticulum chaperone GRP78/BiP and its impact on human disease and therapy
Source: Proc Natl Acad Sci U S A. 2025 Jul 23;122(30):e2412246122. doi: 10.1073/pnas.2412246122 (PMC12318151; doi:10.1073/pnas.2412246122)
Supplement: Supplementary file 1 — Appendix 01 (PDF) [file pnas.2412246122.sapp.pdf]

Supplemental Information

Perspective

Stress-induced translocation of the endoplasmic reticulum chaperone GRP78/BiP and its impact on human disease and therapy

Amy S. Lee

Supplemental Figure S1 and Legend

Supplemental Table S1 and References

Figure S1

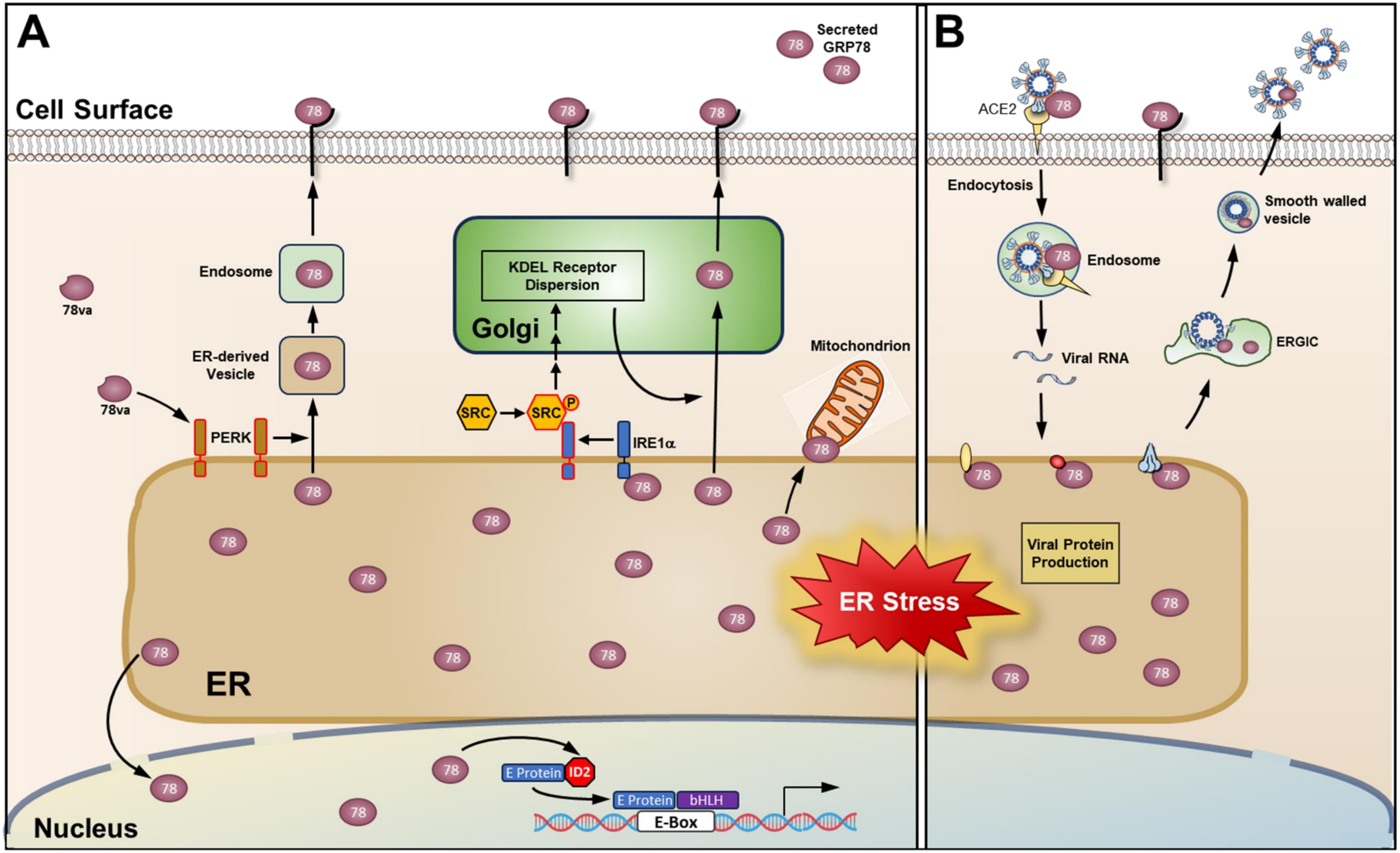

A
B
Secreted GRP78
Cell Surface
Endosome
78va
ER-derived Vesicle
PERK
KDEL Receptor Dispersion
Golgi
SRC
P
IRE1α
Mitochondrion
ER Stress
ER
Nucleus
E Protein
ID2
bHLH
E-Box
ACE2
Endocytosis
Viral RNA
Smooth walled vesicle
ERGIC
Viral Protein Production
78

Supplemental Figure Legend

Figure S1. Summary of ER-stress induced translocation of GRP78 from the ER to various cellular compartments and assuming new functions. (A) Under ER stress, GRP78 can traffic to the cell surface via the ER-Golgi secretory or the endosomal pathway, to the nucleus, to the mitochondria, or be secreted. Alternative splicing generates the 78va isoform, which localizes to the cytosol. The nuclear form of GRP78 sequesters ID2 and regulates transcription. (B) Following viral infection which induces ER stress, GRP78 facilitates viral entry on the cell surface, assists viral protein production in the ER and may be released with the mature virion.

Table S1: Recently identified anti-cancer inhibitors targeting GRP78

Mechanism of action	Agent	Description	Strength	Ref.
**Blocks stress-induced *GRP78* transcription**	IT-139/BOLD-100	A ruthenium-based small molecule therapeutic with a multimodal mechanism of action. Currently undergoing Phase 2 clinical trial for advanced gastrointestinal (GI) cancers in combination with chemotherapy.	Preferentially suppresses GRP78 upregulation in tumor. A first-in-class clinical-stage therapy for advanced cancers. Low systemic toxicity. Dually suppresses cancer and a broad spectrum of viral infections.	1<br>2<br>3
**Blocks stress-induced GRP78 translation**	Oleandrin/PBI-05204	A unique lipid-soluble cardiac glycoside that potently inhibits the Na^+/K^+ ATPase α3 isoform commonly over-expressed in cancer. PBI-05204 containing oleandrin as its active ingredient exhibits safe profile in Phase 1/2 clinical trials in cancer patients.	Oleandrin, a naturally occurring compound, broadly suppresses stress induction of GRP78 at nanomolar concentrations, with enhanced cytotoxicity in malignant cells. Dually suppresses cancer and a broad spectrum of viral infections.	4<br>5<br>6<br>7
**Directly binds GRP78 and blocks its activity**	HA15/BPR001-615	A lead thiazole benzenesulfonamide compound, selectively binds GRP78, suppresses its activity and elicits stress-induced apoptosis and autophagy. Clinical trials are being planned for the treatment of gastrointestinal cancers.	A highly selective GRP78 inhibitor. First-in-class drug candidate overstressing cancer cells. BRP001-615 is being developed for oral use to treat GI cancers in patients screened for GRP78 levels. Dually suppresses cancer and a broad spectrum of viral infections.	8<br>9<br>10
	YUM70	An 8-hydroxyquinone analog, directly binds GRP78, inhibits its activity and triggers ER-stress mediated apoptosis.	Binds GRP78, blocks tumor growth while sparing normal tissues. Dually suppresses cancer and COVID-19.	11<br>12
**Degrades GRP78 protein**	DX2-145	A GRP78 Proteolysis Targeting Chimera (PROTAC) synthesized by incorporating YUM70, a linker and an E3-recruiting ligand. Degrades GRP78 in a proteasome-dependent manner in cancer cells.	This prototype agent demonstrates that GRP78 can be specifically degraded in tumor cells through PROTAC, but more potent derivatives are needed for higher efficacy.	11

Supplemental Table S1 References

1. S. J. Bakewell, *et al.*, Suppression of stress induction of the 78-kilodalton glucose regulated protein (GRP78) in cancer by IT-139, an anti-tumor ruthenium small molecule inhibitor. *Oncotarget* **9**, 29698–29714 (2018).

2. G. M. O'Kane, *et al.*, A phase 2 study of BOLD-100 in combination with FOLFOX chemotherapy in patients with pretreated advanced biliary tract cancer: Efficacy and safety analysis (BOLD-100-001). *JCO* **42**, 4115–4115 (2024).

3. D. S. Labach, *et al.*, The Metallodrug BOLD-100 Is a Potent Inhibitor of SARS-CoV-2 Replication and Has Broad-Acting Antiviral Activity. *Biomolecules* **13**, 1095 (2023).

4. D. P. Ha, *et al.*, Targeting stress induction of GRP78 by cardiac glycoside oleandrin dually suppresses cancer and COVID-19. *Cell Biosci* **14**, 115 (2024).

5. M. T. Roth, *et al*., A Phase II, Single-Arm, Open-Label, Bayesian Adaptive Efficacy and Safety Study of PBI-05204 in Patients with Stage IV Metastatic Pancreatic Adenocarcinoma. *Oncologist* **25**, e1446–e1450 (2020).

6. S. Chakraborty, *et al*., PBI-05204, a supercritical CO2 extract of Nerium oleander, suppresses glioblastoma stem cells by inhibiting GRP78 and inducing programmed necroptotic cell death. *Neoplasia* **54**, 101008 (2024).

7. R. A. Newman, *et al.*, Antiviral Effects of Oleandrin. *J Exp Pharmacol* **12**, 503–515 (2020).

8. M. Cerezo, *et al.*, Compounds Triggering ER Stress Exert Anti-Melanoma Effects and Overcome BRAF Inhibitor Resistance. *Cancer Cell* **29**, 805–819 (2016).

9. W.-J. Shin, D. P. Ha, K. Machida, A. S. Lee, The stress-inducible ER chaperone GRP78/BiP is upregulated during SARS-CoV-2 infection and acts as a pro-viral protein. *Nat Commun* **13**, 6551 (2022).

10. G. Najarro, *et al.*, BiP/GRP78 is a pro-viral factor for diverse dsDNA viruses that promotes the survival and proliferation of cells upon KSHV infection. *PLoS Pathog* **20**, e1012660 (2024).

11. S. Samanta, *et al.,* The Hydroxyquinoline Analogue YUM70 Inhibits GRP78 to Induce ER Stress-Mediated Apoptosis in Pancreatic Cancer. *Cancer Res* **81**, 1883–1895 (2021).

12. D. P. Ha, *et al.,* GRP78 Inhibitor YUM70 Suppresses SARS-CoV-2 Viral Entry, Spike Protein Production and Ameliorates Lung Damage. *Viruses* **15**, 1118 (2023).
